# Structural and Functional Impact of Seven Missense Variants of Phenylalanine Hydroxylase

**DOI:** 10.3390/genes10060459

**Published:** 2019-06-15

**Authors:** Martina Pecimonova, Daniela Kluckova, Frantisek Csicsay, Kamila Reblova, Jan Krahulec, Dagmar Procházkova, Ludovit Skultety, Ludevit Kadasi, Andrea Soltysova

**Affiliations:** 1 Department of Molecular Biology, Faculty of Natural Sciences, Comenius University, Ilkovicova 6, 842 15 Bratislava, Slovakia; mata.pecimonova@gmail.com (M.P.); nela.kluckova@gmail.com (D.K.); jan.krahulec@uniba.sk (J.K.); ludevit.kadasi@uniba.sk (L.K.); 2 Insitute of Virology, Biomedical Research Center, Slovak Academy of Sciences, Dubravska cesta 9, 845 05 Bratislava, Slovakia; frantisek.csicsay@savba.sk (F.C.); viruludo@savba.sk (L.S.); 3 Central European Institute of Technology, Masaryk University, 625 00 Brno, Czech Republic; kristina@monoceros.physics.muni.cz; 4 Department of Pediatrics, Medical Faculty of Masaryk University and University Hospital Brno, Černopolní 9, 625 00 Brno, Czech Republic; Prochazkova.Dagmar@fnbrno.cz; 5 Institute for Clinical and Translational Research, Biomedical Research Center, Slovak Academy of Sciences, Dubravska cesta 9, 845 05 Bratislava, Slovakia

**Keywords:** phenylalanine hydroxylase, phenylketonuria, BH4, functional studies, missense variants

## Abstract

The molecular genetics of well-characterized inherited diseases, such as phenylketonuria (PKU) and hyperphenylalaninemia (HPA) predominantly caused by mutations in the phenylalanine hydroxylase (*PAH*) gene, is often complicated by the identification of many novel variants, often with no obvious impact on the associated disorder. To date, more than 1100 PAH variants have been identified of which a substantial portion have unknown clinical significance. In this work, we study the functionality of seven yet uncharacterized PAH missense variants p.Asn167Tyr, p.Thr200Asn, p.Asp229Gly, p.Gly239Ala, p.Phe263Ser, p.Ala342Pro, and p.Ile406Met first identified in the Czech PKU/HPA patients. From all tested variants, three of them, namely p.Asn167Tyr, p.Thr200Asn, and p.Ile406Met, exerted residual enzymatic activity in vitro similar to wild type (WT) PAH, however, when expressed in HepG2 cells, their protein level reached a maximum of 72.1% ± 4.9%, 11.2% ± 4.2%, and 36.6% ± 7.3% compared to WT PAH, respectively. Remaining variants were null with no enzyme activity and decreased protein levels in HepG2 cells. The chaperone-like effect of applied BH4 precursor increased protein level significantly for p.Asn167Tyr, p.Asp229Gly, p.Ala342Pro, and p.Ile406Met. Taken together, our results of functional characterization in combination with in silico prediction suggest that while p.Asn167Tyr, p.Thr200Asn, and p.Ile406Met PAH variants have a mild impact on the protein, p.Asp229Gly, p.Gly239Ala, p.Phe263Ser, and p.Ala342Pro severely affect protein structure and function.

## 1. Introduction

Phenylketonuria (PKU; OMIM #26160), one of the most frequent disorders of amino acid metabolism in the Caucasian population, affects on average one in 10,000 live births [1]. According to the results of neonatal screening launched in 1985, the PKU incidences in Slovakia (1:5753) [2] and neighbor Czech Republic (1:5378) [3] are very similar. The impairment of phenylalanine hydroxylase (PAH; OMIM #612349), encoded by the *PAH* gene, is in 98% of patients as the major cause of PKU. Due to this impairment, the dietary phenylalanine (Phe) cannot be metabolized properly, which results in the bloodstream. Based on the molar concentration of L-Phe in the blood PKU can be classified as classical PKU, mild PKU, and non-PKU hyperphenylalaninemia [4,5,6]. When left untreated, patients present with mild to severe neurological features, including neurodevelopmental delay, intellectual disability, seizures, and microcephaly, or they can develop psychiatric disorders along with behavioral, emotional, and social problems as well [7]. Early restrictive diet intervention and its lifelong strict compliance are essential for the prevention of these symptoms [4,5].

For the proper enzymatic function, i.e., the catalysis of para-hydroxylation of L-Phe and subsequent generation of L-Tyr, PAH is assembled in the tetrameric formation and requires tetrahydrobiopterin (BH4) as a cofactor, free molecular oxygen, and non-heme iron [8]. 

To date, more than 1100 PAH variants have been identified and recorded in the PAHvdb database [9] of which approximately 50% are missense mutations often with unknown clinical significance. The common cause of PKU is mutation-driven protein instability that affects PAH resulting in subsequent protein turnover [10,11] that in some cases can be rescued, at least partially, by molecular chaperones. The severity of PAH variants range from mild to severe null mutations. PKU patients carrying mild PAH variants that exert residual enzymatic activity, and some null variants lacking this activity, can profit from BH4 supplementation as it acts as a pharmacological chaperone that stabilizes tertiary and/or quaternary PAH structure lowering its degradation rate in the cell [12,13,14,15].

In this study, we performed the functional analyses of seven novel PAH variants first discovered in the Czech population [16] to determine their potential clinical significance. The p.Asn167Tyr, p.Thr200Asn, p.Asp229Gly, p.Gly239Ala, p.Phe263Ser, p.Ala342Pro, and p.Ile406Met PAH variants were expressed in prokaryotic and eukaryotic expression systems and assayed in vitro to investigate their specific activity, oligomeric pattern, and the mutant PAH protein stability in the presence of sepiapterin, which is the BH4 precursor, as well as GroEL/ES bacterial chaperones.

## 2. Materials and Methods 

### 2.1. In Silico Analyses 

Two web-based applications for the impact prediction of amino acid substitution on protein function, i.e., Meta-SNP [17] and PredictSNP [18] were employed. Both predictors combine several existing programs for the resulting variant classification; Meta-SNP [17] integrated four different programs and PredictSNP [18] uses the combination of six different programsAs an input, NCBI reference protein sequence NP_000268.1 in FASTA format was used.

Since p.Asn167Tyr, p.Thr200Asn, p.Asp229Gly, p.Phe263Ser, and p.Ile406Met variants were analyzed in silico in our previous study [16], we performed modeling only for p.Gly239Ala and p.Ala342Pro based on experimental X-ray structures.

We used our previous 3D model of human tetrameric PAH structure, which was assembled using the Pymol program (https://pymol.org/2/) based on a dimer determined by X-ray crystallography (Protein Database (PDB) ID: 2PAH). For more details about the building of this model see our previous study [19]. We also used new experimental tetrameric rat PAH structure ([20]: First structure of full-length mammalian phenylalanine hydroxylase reveals the architecture of an autoinhibited tetramer; PDB ID: 5DEN).

For two studied variants we calculated the buriedness of the WT PAH AA in the monomer forming dimeric structure and also in subunits forming the tetrameric structures. Solvent accessibility of the residues in the protein structure was calculated using the STRIDE program [21] and divided by the total surface area of the residue [22]. This value corresponds to the relative accessible surface area (RSA). A residue is considered buried if the RSA is <10%. It can be expected that replacing a buried AA is more likely to be associated with a structural defect, especially when charge, polarity, and volume are changed upon a mutation. Volume change upon a mutation ≥20 Å3 associated with the large to small substitution was considered destabilizing.

### 2.2. Mutant PAH Recombinant Constructs Preparation 

The pMAL-MBP-c2 vector for bacterial expression containing human (WT) PAH complementary DNA (cDNA) in fusion with maltose-binding protein (MBP) was kindly provided by Prof. Lourdes R. Desviat from the Centro de Biologia Molecular, Autonomous University of Madrid, Spain. Nucleotide substitutions NM_000277.2:c.499A>T, NM_000277.2:c.599C>A, NM_000277.2:c.686A>G, NM_000277.2:c.716G>C, NM_000277.2:c.788T>C, NM_000277.2:c.1024G>C, and NM_000277.2:c.1218A>G were inserted into the pMAL-PAH construct using Phusion Site-Directed Mutagenesis Kit (Thermo Fisher Scientific, Waltham, Massachusetts, USA) and the mutagenic primers are listed in Table 1.

Recombinant plasmids for the testing in eukaryotic expression system were prepared by cloning of PAH cDNA sequence into pFLAG-CMV vector. Inserts were obtained by amplification of WT and mutant pMAL-PAH constructs using cloning primers 5′-TTTTGCGGCCGCGATGTCCACTGCGGTCCTGG-3′ and 5′-AAAAGTCGACGGCTTTACTTTATTTTCTGGAG-3′ carrying NotI and SalI restriction sites [23] allowing the subsequent cloning into the vector. Successful mutagenesis, cloning as well as exclusion of unintended mutations was verified by DNA sequencing.

### 2.3. Human PAH Expression in Escherichia coli and Protein Purification

Human PAH proteins were expressed in XL1 blue cells, with or without co-transformation of pGroELS plasmid coding GroEL and GroES bacterial chaperones. The expression was induced by the addition of 100 mM IPTG (Isopropyl-β-D-thiogalactoside) simultaneously with 0.2 mM FAS (ferrous-ammonium-sulfate) to cultivation medium at optical density (OD) = 0.5. PAH proteins were expressed for 18 hours at 37 °C. Cells were collected and sonicated in the buffer comprised of 200 mM NaCl, 20 mM HEPES ((4-(2-hydroxyethyl)-1-piperazineethanesulfonic acid), and 0.2 mM Pefabloc, pH = 7.0 at 4 °C. PAH proteins were subsequently purified using the ÄKTA avant 25 system (GE Healthcare Life Sciences Marlborough, Massachusetts, USA) by affinity and size-exclusion chromatography using MBPTrapTM HP 1 mL column (GE Healthcare Life Science) and Superdex 200 HR 10/30 column (GE Healthcare Life Sciences), respectively. For the purification procedure, the column buffer containing 200 mM NaCl, 20 mM HEPES, pH = 7.0 was used. The protein elution was performed using 10 mM maltose admixed with the column buffer. PAH tetrameric forms were collected, if absented the mixture of PAH oligo- and dimers was collected, and spectrophotometrically quantified using Quick StartTM Bradford Protein Assay (Bio-Rad, Hercules, CA, USA).

### 2.4. PAH Functional Assay 

PAH functional assay was performed as previously described by Martinez et al. (1995) [24] at the constant temperature of 25 °C. 1 μg of PAH protein was pre-incubated with 10 mM L-Phe in the buffer containing 0.1 M NaHEPES and 1 mg/ml catalase for four minutes. After the addition of 100 μM FAS, the reaction mixture was incubated for one minute. The conversion of L-Phe to L-Tyr was initiated by the addition of 75 μM BH4 diluted in 5 mM DTT and stopped after one minute by the addition of the 1% w/v acetic acid in ethanol. 

Tyrosine and phenylalanine were separated by high-performance liquid chromatography (HPLC) Agilent 1100 series (Agilent, Santa Clara, CA, USA) in an analytical column Zorbax XDB-C18; 150 × 4.6 mm; 5 μm. The mobile phase was isocratic with the column temperature set at 30 °C. Signals were detected by a fluorescence detector with an excitation wavelength of 210 nm and analyzed by Chemstation software (Agilent). Residual enzymatic activity was expressed percentually as the quotient of the molar concentration of L-Tyr produced during the reaction catalyzed by mutant PAH protein compared to that of WT PAH.

### 2.5. Eukaryotic Expression

Human liver hepatocellular cells HepG2 were cultivated in the Eagle’s Minimum Essential Medium (EMEM) (Sigma-Aldrich, St. Louis, MO, USA) supplemented with 10% Fetal Bovine Serum (FBS) (Sigma-Aldrich) in 6-well plates; each tested pFLAG-PAH variant was cultivated in duplicate. The cells were transfected at the confluency of 70%–90% by 2.5 μg of WT and mutant pFLAG-PAH using Lipofectamine^®^2000 (Thermo Fisher Scientific) and incubated overnight. Subsequently, the transfecting mixture was replaced with the fresh EMEM supplemented with 10% FBS. At this time, one of the duplicates of each tested WT/mutant PAH containing transfected cells was treated with 100 μM sepiapterin (Sigma-Aldrich). The cells were collected 36 h after the transfection and lysed on ice in the NP-40 buffer containing NP-40 lysis buffer containing 150 mM sodium chloride, 1.0% Nonidet P-40, and 50 mM Tris, pH 8.0. The protein concentration was determined spectrophotometrically using Quick StartTM Bradford Protein Assay (Bio-Rad).

### 2.6. Western Blot Analysis

Mutated PAH proteins (10 ng each) were separated on 10% SDS-PAGE (sodium dodecyl sulfate-polyacrylamide gel electrophoresis) and blotted on PVDF (polyvinylidene fluoride) membrane. PAH proteins were detected using Monoclonal ANTI-FLAG^®^ M2 antibody produced in mice (Sigma Aldrich) and Anti-Mouse IgG (H+L) HRP (Horseradish peroxidase) Conjugate (Promega, Fitchburg, WI, USA). Chemiluminescent signal detection was performed on ImageQuant LAS 500 (GE Healthcare Life Science) using Immobilon Western Chemiluminescent HRP Substrate (Merck Millipore, Burlington, MA, USA). Protein bands intensity was quantified using GelQuant.NET software (http://biochemlabsolutions.com/GelQuantNET.html). 

### 2.7. Statistical Analysis

Each of the presented results was calculated as a mean of multiple experiment outputs including standard deviation. Statistical differences between multiple comparisons and *p*-value determination were performed using a Student’s *t*-test with the two-tailed distribution and two-sample equal variance (homoscedastic).

## 3. Results

### 3.1. In Silico Analysis

Meta-SNP and PredictSNP approaches integrating several existing programs for the more accurate prediction of the impact of amino acid substitution were used to evaluate the studied variants. p.Asn167Tyr, p.Asp229Gly, p.Gly239Ala, p.Phe263Ser, and p.Ala342Pro were classified as pathogenic and p.Ile406Met as neutral by both consensus tools. p.Thr200Asn was characterized as the disease causing variant by Meta-SNP but it was determined as neutral by PredictSNP. The results are summarized in Table 2 and include the overall evaluation from individual programs employed by both approaches.

**Figure 1:**
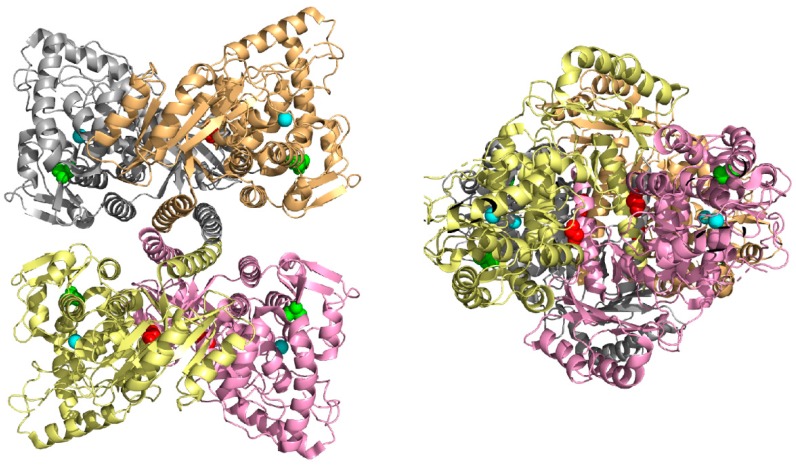
Top view (left) and side view (right) on the X-ray tetrameric rat PAH structure with color monomers. Gly239 is highlighted in sphere representation in red, Ala342 is in green, and Fe ion in the active site is in cyan.

Two PAH variants p.Gly239Ala and p.Ala342Pro were subjected to the in silico structural modeling based on tetrameric human and rat PAH structures. Analysis of other variants was carried out in our previous study [16]. In the PAH monomeric form, Gly239 is partially exposed to solvent (buriedness is 46%), hence the substitution for larger alanine should have no impact on protein structure. However, in the PAH tetrameric form Gly239 is more buried suggesting a possible deleterious impact on forming tetramers. In particular, buriedness of Gly239 in subunits A-D forming human tetrameric structure is 18%, 11%, 19%, and 11% (due to symmetry buriedness in subunits A,C, and B,D is almost identical), in the rat tetrameric structure the Gly239 is even more buried, buriedness of Gly239 in subunits A-D is 0%, 14%, 16%, and 3% (Figure 1). In the case of p.Ala342Pro PAH variant, Ala342 is entirely buried in the monomeric PAH structure and all subunits of tetrameric structures (Figure 1). The substitution of alanine for larger proline (difference in volume is 24 Å^3^) could have an impact on protein structure. In addition, Ala342 is positioned at the end of β-sheet structure and it is known that substitution of any AA to proline in alpha-helical or beta-sheet structures is considered destabilizing [25], suggesting a possible pathogenic impact on the PAH protein.

### 3.2. Human PAH Expression in Escherichia coli and Oligomeric Pattern

Human WT and mutant PAH-MBP fusion proteins expressed in XL1 blue cells were column purified using affinity and subsequent size-exclusion chromatography. Specific pattern of expressed WT PAH comprised of 29.9% ± 11.3% of oligomers, 58.7% ± 15.2% of catalytically active tetramers and 11.4% ± 9% of dimers and co-cultivation with GroEL/ES bacterial chaperones resulted in insignificant increase in tetramers up to 60.5% ± 2.9%, followed with 34.8% ± 3.5% of oligomers and 4.6% ± 3.7% of dimers.

The protein expression was detected in all tested PAH variants forming predominantly high molecular aggregated oligomers. In general, two patterns were observed in PAH variants expression with neither of them following the pattern of WT PAH. p.Asp229Gly, p.Gly239Ala, p.Phe263Ser, and p.Ala342Pro PAH variants fell into the group that predominantly formed aggregated oligomers followed with dimers and very little or no tetramers with the values of 4.1% ± 3.7%, 1.3% ± 2.2%, 5.5% ± 4.8%, and 0.0% ± 0.0% respectively. p.Asn167Tyr, p.Thr200Asn, and p.Ile406Met PAH variants followed the second pattern, where catalytically active tetramers represented approximately a third (33.2% ± 5.8%, 40.5% ± 6.1%, and 34.2% ± 4.5% respectively) of the total purified protein, oligomeric forms counts for approximately 50% and dimers formed from 10.5% to 20.2%.

Co-expression of all PAH variants in the presence of GroEL/ES bacterial chaperones increased the tetramer formation and decreased the dimer portions. In p.Asn167Tyr, p.Thr200Asn, and p.Ile406Met PAH variants tetramer portions increased to 38.8% ± 4.2%, 43.8% ± 3.6%, and 37.7% ± 7.4%, respectively. The higher tetramer ratio was also detected in p.Asp229Gly, p.Gly239Ala, p.Phe263Ser, and p.Ala342Pro PAH variants where they represented 10.2% ± 5.6%, 5.5% ± 4.8%, 11.0% ± 4.7%, and 3.8% ± 4.0%, respectively. However, this increase did not reach the statistical significance in any of the PAH mutants. Three independent column purifications for all PAH proteins were performed. The average percentage ratio of individual PAH protein forms obtained after size-exclusion chromatography are summarized in Table 3.

### 3.3. PAH Functional Assay

Enzymatic activity of each PAH protein was functionally tested in nine independent reactions, i.e., three assays for each protein purification of the given mutant. p.Asn167Tyr and p.Thr200Asn PAH proteins showed residual enzymatic activity similar to WT PAH (97.8% ± 27.1% and 92.4% ± 25.9%, respectively) and p.Ile406Met exerted 84.6% ± 48.4% of WT PAH activity. Interestingly, the residual activities of p.Thr200Asn and p.Ile406Met PAH variants co-expressed in the presence of GroEL/ES chaperones decreased to 86.3% ± 24.7% and 76.7% ± 14.5%, respectively, while that of p.Asn167Tyr PAH variant increased nonsignificantly to 108.0% ± 27.0%. No residual activities of p.Asp229Gly, p.Gly239Ala, p.Phe263Ser, and p.Ala342Pro PAH variants were detected without GroEL/ES chaperones. While residual activity of p.Asp229Gly PAH variant remained null when co-expressed with GroEL/ES, the activities of p.Gly239Ala, p.Phe263Ser, and p.Ala342Pro PAH variants increased to 0.9% ± 1.5%, 0.9% ± 2.2%, and 9.9% ± 0.7% compared to WT PAH, respectively. The complete results of PAH residual activities are listed in Table 3.

### 3.4. Western Blot Analysis

**Figure 2:**
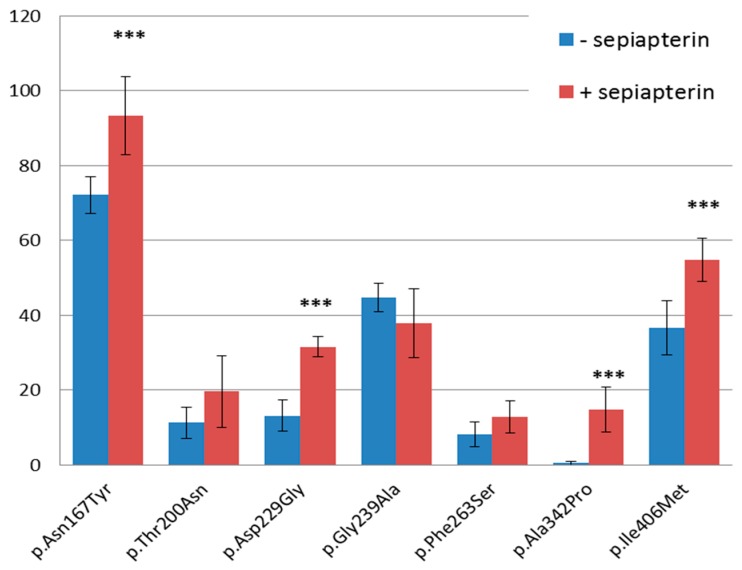
Mutant PAH protein levels compared to WT PAH expressed in HepG2 cells in the presence and absence of BH-4 precursor sepiapterin. The results were calculated as a mean of three independent Western blot analyses performed from three independent PAH expressions in HepG2 cells. *** statistically significant level changes with the *p*-value < 0.0001 between mutated proteins expressed in the presence and absence of sepiapterin.

WT and mutant PAH transient expression in HepG2 cells were performed in the presence as well as the absence of BH-4 precursor sepiapterin. As shown in Figure 2, western blot analysis revealed decreased protein levels of each tested PAH mutant cultivated under both conditions compared to WT PAH. All but one PAH variant, namely p.Gly239Ala, showed higher protein levels when cultivated in the medium treated with the sepiapterin compared to those untreated, with the significant increase in protein level observed in p.Asn167Tyr, p.Asp229Gly, p.Ala342Pro, and p.Ile406Met PAH variants with the *p*-values of 0.00053, 1.66 × 10^−7^, 1.05 × 10^−5^, and 0.000843, respectively. The overall results were calculated from three independent Western blot analyses performed from three independent PAH expressions in HepG2 cells.

## 4. Discussion

To date, more than 1100 PAH variants, associated with a group of disorders ranging from mild hyperphenylalaninemia to severe classical PKU, have been discovered and new ones are still being identified [10]. The determination of the clinical significance of unknown PAH variant provides insight into genotype–phenotype correlations and helps deploy the most optimal and personalized treatment, especially in connection with BH4 responsiveness [26].

The variant interpretation is a complex process, which relies upon various approaches and results in the variant classification: Pathogenic, likely pathogenic, uncertain significance, likely benign, or benign. Based on the guidelines of the American College of Medical Genetics and Genomics and the Association for Molecular Pathology for variant interpretation, the results of well-established in vitro functional studies represent strong evidence either of pathogenicity or benign impact [27]. Herein, we performed functional analysis to specify protein expression levels, the oligomeric pattern and enzymatic activity, of seven PAH missense variants, namely p.Asn167Tyr, p.Thr200Asn, p.Asp229Gly, p.Gly239Ala, p.Phe263Ser, p.Ala342Pro, and p.Ile406Met, previously reported in Czech PKU patients [16].

The expression of all tested PAH variants followed two patterns: i) PAH variants formed predominantly high molecular aggregated forms followed with lower levels of dimers and very little or no tetramers, and ii) PAH variants formed aggregated oligomers representing approximately the half of expressed protein followed with more than a third of catalytically active tetramers and low dimer levels. Even when co-expressed with GroEL/ES bacterial chaperones we observed an insignificant increase of tetramer formation and decreased dimer portion. None of these two patterns correlates with the expression profile of WT PAH, which consists of approximately 27% of oligomers, 60% of tetramers, and 13% of dimers [28]. This is consistent with our findings suggesting the possible milder to severe deleterious impact of tested variants on PAH structure/function. The oligomeric pattern correlated well with the measured residual enzyme activities of tested PAH variants expressed in XL1 blue cells, which were either null for those lacking tetramers (i.e., p.Asp229Gly, p.Gly239Ala, p.Phe263Ser, and p.Ala342Pro), or slightly reduced ranging from 83.3% ± 17.5% to 97.8% ± 27.1% compared to WT PAH (i.e., p.Asn167Tyr, p.Thr200Asn, and p.Ile406Met). The co-expression with GroEL/ES bacterial chaperones resulted in an increase of the residual enzyme activity for p.Asn167Tyr, p.Gly239Ala, p.Phe263Ser, and p.Ala342Pro, which may indicate that these substitutions affect proper PAH folding. Results of the PAH functional assay are in agreement with the outputs of the in silico analysis using Meta-SNP and PredictSNP tools. A predicted deleterious effect by both tools was detected for p.Asn167Tyr, p.Asp229Gly, p.Gly239Ala, p.Phe263Ser, and p.Ala342Pro PAH variants. In the case of p.Thr200Asn, Meta-SNP determined this variant as deleterious while PredictSNP predicted this variant to be neutral. Further, the neutral effect of p.Ile406Met predicted by both in silico predictors was not confirmed by our in vitro experiments as decreased enzyme activity compared to WT PAH was detected.

Moreover, the expression analysis performed on human hepatic cell line HepG2 revealed reduced PAH protein levels in all PAH variants. A significant increase in variant PAH protein level after sepiapterin treatment, which is a PAH cofactor that acts as a pharmacological chaperone, was observed for p.Asn167Tyr, p.Asp229Gly, p.Ala342Pro, and p.Ile406Met. Since BH4-responsiveness was observed in patients presented with all the spectra of PKU phenotypes including severe cPKU [29,30,31,32], the individuals carrying one of these variants could profit from the BH4-supplementation.

The potential BH4-responsiveness of p.Asn167Tyr was supported by findings reported by Jeannesson-Thivisol et al. (2015) who tested the BH4-responsiveness of p.Asn167Ile, another substitution in the same position, with a positive outcome in a French patient concluded the allele to be BH4-responsive [31]. Zurfluh et al. (2008) proposed that mild PKU phenotypes have a better response on BH4-supplementation [33], which was later supported by subsequent studies [30,31,34,35,36,37]. Zhu et al. (2017) found out that 89.58% of BH4-responders had mild phenotypes and the remaining 10.42% were presented with cPKU [38]. Although knowing the patient’s complete genotype is considered to be the BH4-responsiveness predictor [39], the interallelic complementation appears to affect the resultant phenotype and response on BH4-supplementation [40,41]. Therefore, the BH4-loading test and its correlation with the patient’s genotype is still the most reliable approach to determine the BH4-responsiveness [42].

A mild impact of p.Asn167Tyr, p.Thr200Asn, and p.Ile406Met PAH variants were also described based on molecular dynamics simulations [16]. Although p.Thr200Asn expressed lower protein levels in HepG2 cells (11.2% vs. 19.6% with sepiapterin) suggesting a higher degradation rate, its residual activity observed in vitro is similar to WT PAH (92.4% ± 25.9% compared to WT PAH) and probably compensates for these low protein levels. Overall, our results of in vitro testing of p.Asn167Tyr, p.Thr200Asn, and p.Ile406Met PAH variants are consistent with patients’ non-PKU HPA phenotypes without low-Phe dietary compliance, all compound heterozygotes carrying genotypes p.[Asn167Tyr];[Arg408Trp], p.[Thr200Asn];[Arg243*], p.[Ile406Met];[Arg408Trp], or p.[Ile406Met];[Ex5del] [16]. Genotype p.[Ile406Met];[Arg408Trp] was also identified in Slovak non-PKU HPA patient. The concordant results were obtained for p.Gly239Ala, p.Phe263Ser, and p.Ala342Pro PAH variants, as well. All reported patients carrying these variants in a compound heterozygous state p.[Gly239Ala];[Arg243*], p.[Phe263Ser];[c.1066-11G>A], or p.[Ala342Pro];[Pro281Leu] presented with cPKU [14]. p.Phe263Ser was also identified in combination with a p.Tyr166* allele, recorded in the ClinVar database (https://www.ncbi.nlm.nih.gov/clinvar/) as pathogenic, in a Chinese patient presented with classic PKU [43]. The pathogenic effect of p.Phe263Ser PAH variant was described in the previous study [16]. In more detail, Phe263 is localized in the active site and the disruption of the important stacking interaction with Phe294 caused by the substitution for serine most probably results in protein destabilization and impaired PAH activity. The suggested deleterious impact of the p.Gly239Ala substitution on tetramer assembly correlates well with results obtained from size exclusion chromatography, which evidenced impaired tetramer formation where only 1.3% ± 2.2% of total purified protein was detected. In silico modeling of p.Ala342Pro showed that the residue Ala342 is buried in the protein and its substitution for proline most probably impairs the local protein structure suggesting a possible pathogenic impact on the PAH protein.

As reported above, the results are concordant with patients phenotypes were observed in all tested PAH variants except for p.Asp229Gly. Although Reblova et al. (2013) [16] reported that the substitution of aspartic acid for uncharged glycine abolished the salt bridge with Arg176, based on the molecular dynamics simulation they assumed this interaction is not essential in the WT PAH conformation supported by the patient with the genotype p.[Arg176Leu];[Arg408Trp] and the non-PKU HPA phenotype [44]. This correlates with the patient’s non-PKU HPA phenotype carrying p.[Asp229Gly];[Arg408Trp]. Our functional analysis of p.Asp229Gly revealed no residual activity even when co-expressed with GroEL/ES chaperones, which indicates a probable pathogenic effect of this variant in agreement with ACMG criteria fulfilling the following two moderate and two supporting evidence of pathogenicity [27]. The discrepancy between our findings and the patient’s phenotype might indicate the involvement of other additional genetic factors or PKU-modifying genes [45,46].

In conclusion, we performed functional studies of seven yet uncharacterized missense PAH variants complemented by in silico predictions. Following ACMG standards for variant classification [27], solely in silico analysis of mutated proteins is insufficient in the assessment of clinical significance due to the fact that the prediction accuracy of the best prediction tools is ca. 80% [47]. This highlights the necessity of using other approaches including in vitro studies. Taken together, our results of both the functional testing and expression analysis suggest a severe impact of p.Asp229Gly, p.Gly239Ala, p.Phe263Ser, and p.Ala342Pro PAH variants, while a mild effect of p.Asn167Tyr, p.Thr200Asn, and p.Ile406Met variants on the protein structure/function. We also identified the potential candidates profiting from BH4-supplementation carrying either a p.Asn167Tyr or p.Ile406Met PAH variant. Overall, when translated to clinical practice, our findings could be useful in adjusting the proper personal treatment of PKU patients by clinicians.

## Figures and Tables

**Table 1:** The primers used for PAH mutants preparation using site-directed mutagenesis. Bolded nucleotide represents the substituted base.

PAH Mutant		Primer Sequence (5′→3′)
p.Asn167Tyr	c.499A > T	CATTGCC**T**ACTACTACCGCC
		TCAGCAAACTGCTTCCGTC
p.Thr200Asn	c.599C > A	CTTGTATAAAA**A**CCATGCTTGC
		GACTTCAGAGTCTTGAACACTGT
p.Asp229Gly	c.686A > G	CAGCTGGAAG**G**CGTTTCTCA
		GGGAATGTTATCTTCATGGAAGCC
p.Gly239Ala	c.716G > C	ACTTGCACTG**C**TTTCCGCCT
		CTGCAGGAACTGAGAAACGTCTTCC
p.Phe263Ser	c.788T > C	TTCCGAGTCT**C**CCACTGCACA
		GGCCAGGCCACCCAAGAAAT
p.Ala342Pro	c.1024G > C	CTCCATAAAG**C**CATATGGTGCTG
		TCTCCTTGTTTGCAGAGCCC
p.Ile406Met	c.1218A > G	TGCCACAAT**G**CCTCGGCCCT
		GCAAAGTTCCTTACTTTCTCCTTGGCATCATT

**Table 2:** The results of in silico PAH variant pathogenicity prediction by Meta-SNP and PredictSNP. “Neutral” referred to a neutral variant, “Disease” and “Deleterious” to disease-causing variant. Variant was predicted to be disease-causing when outputs of Meta-SNP were >0.5. The percentages express the expected prediction accuracies of PredictSNP.

PAH Variant	Meta-SNP	PredictSNP
p.Asn167Tyr	Disease	Deleterious
	0.542	51%
p.Thr200Asn	Disease	Neutral
	0.533	65%
p.Asp229Gly	Disease	Deleterious
	0.542	72%
p.Gly239Ala	Disease	Deleterious
	0.752	87%
p.Phe263Ser	Disease	Deleterious
	0.826	87%
p.Ala342Pro	Disease	Deleterious
	0.824	87%
p.Ile406Met	Neutral	Neutral
	0.414	65%

**Table 3:** The percent representation of individual assemblies of purified (WT) and mutant PAH proteins after their expression in *Escherichia coli* in the presence and absence of GroEL/ES chaperones determined as a mean of three independent column purifications. The residual activity represents the percentage ± standard deviation (SD) of PAH enzymatic activity compared to WT PAH activity and was calculated as a mean of nine independent functional assays.

PAH Variant	− GroEL/ES				+ GroEL/ES			
	Oligomers (%)	Tetramers (%)	Dimers (%)	Residual Activity (%)	Oligomers (%)	Tetramers (%)	Dimers (%)	Residual Activity (%)
p.Asn167Tyr	46.6 ± 11.2	33.2 ± 5.8	20.2 ± 5.8	97.8 ± 27.1	52.7 ± 2.0	38.8 ± 4.2	8.5 ± 4.1	108.0 ± 27.0
p.Thr200Asn	49.0 ± 3.5	40.5 ± 6.1	10.5 ± 6.3	92.4 ± 25.9	51.7 ± 2.6	43.8 ± 3.6	4.5 ± 1.2	86.3 ± 24.7
p.Asp229Gly	76.5 ± 13.1	4.1 ± 3.7	19.3 ± 13.8	0	79.7 ± 8.4	10.2 ± 5.6	10.1 ± 2.9	0
p.Gly239Ala	65.9 ± 7.0	1.3 ± 2.2	32.8 ± 5.8	0	80.5 ± 8.2	5.5 ± 4.8	14.0 ± 3.4	0.9 ± 1.5
p.Phe263Ser	67.4 ± 15.4	5.5 ± 4.8	27.1 ± 10.8	0	75.4 ± 7.8	11.0 ± 4.7	13.6 ± 3.7	0.9 ± 2.2
p.Ala342Pro	78.0 ± 11.1	0	22.0 ± 11.1	0	81.8 ± 3.9	3.8 ± 4.0	15.5 ± 7.9	9.9 ± 0.7
p.Ile406Met	50.3 ± 8.9	34.2 ± 4.5	15.6 ± 4.4	83.3 ± 17.5	55.5 ± 9.9	37.7 ± 7.4	6.9 ± 2.3	76.7 ± 14.5
